# 419 A CTS team approach to identifying thematic constructs related to kratom use during pregnancy and breastfeeding: A qualitative analysis of social media posts – ADDENDUM

**DOI:** 10.1017/cts.2023.592

**Published:** 2023-07-26

**Authors:** Carolin C. Hoeflich, Michelle A. Kuntz, Christopher R. McCurdy, Catherine W. Striley

The above abstract [1] published without the full text of the METHODS/STUDY POPULATION section. The full section should read as follows:

METHODS/STUDY POPULATION: Pregnancy- and breastfeeding-related keywords are being used to extract posts and selected metadata from the following subreddit communities: r/kratom, r/quittingkratom, r/pregnant, and/or r/breastfeeding. After the removal of duplicate posts, posts written in a non-English language and those that state in the post text and/or title that they were published by minors (<18 years old) are being excluded. Posts that do not describe human kratom use during pregnancy or breastfeeding are also being eliminated. Inductive thematic analyses will be performed by two independent reviewers, with coding discordance settled by a third reviewer.

The original abstract has been corrected online to rectify this omission.
